# Genetic variability of environmental sensitivity revealed by phenotypic variation in body weight and (its) correlations to physiological and behavioral traits

**DOI:** 10.1371/journal.pone.0189943

**Published:** 2017-12-18

**Authors:** Delphine Lallias, Edwige Quillet, Marie-Laure Bégout, Benoit Aupérin, Hooi Ling Khaw, Sandie Millot, Claudiane Valotaire, Thierry Kernéis, Laurent Labbé, Patrick Prunet, Mathilde Dupont-Nivet

**Affiliations:** 1 GABI, INRA, AgroParisTech, Université Paris-Saclay, Jouy-en-Josas, France; 2 Laboratoire Ressources Halieutiques, Ifremer, Place Gaby Coll, L’Houmeau, France; 3 INRA, UR 1037 Laboratoire de Physiologie et Génomique des Poissons, Campus de Beaulieu, Rennes, France; 4 INRA, UE 0937 PEIMA (Pisciculture Expérimentale INRA des Monts d’Arrée), Sizun, France; University of Tasmania, AUSTRALIA

## Abstract

Adaptive phenotypic plasticity is a key component of the ability of organisms to cope with changing environmental conditions. Fish have been shown to exhibit a substantial level of phenotypic plasticity in response to abiotic and biotic factors. In the present study, we investigate the link between environmental sensitivity assessed globally (revealed by phenotypic variation in body weight) and more targeted physiological and behavioral indicators that are generally used to assess the sensitivity of a fish to environmental stressors. We took advantage of original biological material, the rainbow trout isogenic lines, which allowed the disentangling of the genetic and environmental parts of the phenotypic variance. Ten lines were characterized for the changes of body weight variability (weight measurements taken every month during 18 months), the plasma cortisol response to confinement stress (3 challenges) and a set of selected behavioral indicators. This study unambiguously demonstrated the existence of genetic determinism of environmental sensitivity, with some lines being particularly sensitive to environmental fluctuations and others rather insensitive. Correlations between coefficient of variation (CV) for body weight and behavioral and physiological traits were observed. This confirmed that CV for body weight could be used as an indicator of environmental sensitivity. As the relationship between indicators (CV weight, risk-taking, exploration and cortisol) was shown to be likely depending on the nature and intensity of the stressor, the joint use of several indicators should help to investigate the biological complexity of environmental sensitivity.

## Introduction

Phenotypic plasticity is the ability of a given genotype to produce different phenotypes in response to varying environments (e.g. [1]). It may be adaptive, neutral or maladaptive [2–4] whether the phenotypic change leads to a more optimal phenotype with favorable characteristics or not. Adaptive phenotypic plasticity is thus a key component of the ability of organisms to cope with changing environmental conditions [5].

In order to investigate the genetic factors underlying phenotypic plasticity, two main experimental approaches are traditionally used. Evolutionary-oriented approaches involve the testing of several genetic groups (e.g. families, strains or populations) in several environments, the level of phenotypic plasticity being assessed through genotype-environment interactions [2] or reaction norms [6]. In animal breeding-oriented approaches, experiments are usually carried out in a single (macro)environment, the level of phenotypic plasticity being assessed through the scale of the within-group phenotypic residual variance in response to microenvironmental fluctuations [7] i.e. sensitivity to environment. As shown by Debat and David [8], these approaches may not refer exactly to the same concept (phenotypic plasticity, canalization, developmental stability) but are all linked to sensitivity to the environment.

Fish have been shown to exhibit a substantial level of phenotypic plasticity in response to a variety of abiotic (e.g. [9–11]) and biotic (e.g. [12,13]) factors. Furthermore, phenotypic plasticity in the context of adaptation to changing environmental conditions is particularly relevant for fish. Indeed, poikilothermy and close interactions with their living medium make them highly dependent on variation in water characteristics, including temperature, dissolved oxygen, pH, pollutants or pathogens. Management practices like transport, handling, confinement or dietary shifts can also severely affect farmed fish. In the context of animal farming, sensitivity to the environment can be seen as an important component of robustness. Robustness has been defined as the ability to combine production performances together with high resilience to external stressors [14]. Therefore, genotypes that have the ability to maintain a given (productive) phenotype in a range of environments, i.e. that have a low sensitivity to changes in biotic and abiotic environment, are highly desirable. Organisms characterized by low sensitivity to environmental stress can either have biological sensors not sensitive to the stress; or have a good ability to cope with the stress.

Generally, physiological and behavioral indicators are used to assess the sensitivity of a fish to environmental stressors and hence its ability to cope with changes. In fish, as in other animals, the most commonly used physiological indicators include the primary (endocrine) response to the stressor (plasma catecholamines, cortisol and ACTH) and the secondary (metabolic) response with variables or traits like plasma glucose, hematocrit, hemoglobin, plasma protein and condition factor [15]. Behavioral indicators of sensitivity to environmental stress in farmed fish include foraging behavior (feeding motivation and feed intake), ventilatory activity, aggression, individual or group swimming behavior in different situations, exploratory behavior and food-anticipatory activity [16].

To improve fish welfare [17], including robustness in selective breeding schemes would allow balancing genetic change in production potential with genetic change in sensitivity to the environment [18]. It is therefore of prime importance to understand the genetic determinism of the sensitivity to the environment. Are some genotypes more sensitive to their environment than others? Which factors influence the sensitivity to the environment? Are the different indicators of environmental sensitivity consistent? In animal breeding-oriented approaches, the genetics of environmental sensitivity is an active field of research, with statistical models [19–21] being developed to estimate genetic variability of residual variance. However, those models rely on family structures, and need high number of large-size families to obtain precise estimates. This leads to large and costly experiments, especially when one wants to measure many and/or not easy-to-measure traits. In aquaculture species, there are only a few studies on the genetics of environmental sensitivity (reviewed in [22]).

The use of original biological resources, i.e. isogenic lines, offers a powerful alternative to overcome the difficulties encountered with standard populations when exploring the genetic determinism of sensitivity to environment. Isogenic lines of rainbow trout *Oncorhynchus mykiss* have been produced after two successive generations of gynogenetic reproduction [23]. Within each line, all fish are expected to be genetically identical, while the different lines are a sample of the genetic variability existing in the population they are derived from. Because there is no within-line genetic variability, the within-line (among fish within tanks) phenotypic variance is a direct estimator of the environmental variance, i.e. the environmental sensitivity of the line genotype [24]. This animal material thus allows easy access to genetic variability of environmental variance with experiments of reasonable size.

Using those isogenic lines, we demonstrated in a previous study the existence of a genetic determinism of the within-line phenotypic variability of body weight at early stages [24], i.e. individual genotypes (lines) have the ability to express more or less homogeneous phenotype when kept in the same environment. Body weight was chosen as a trait expected to be integrative of the whole life history of each individual fish and to be representative of its ability to cope with the different environmental events it encountered. One of the aims of the present study was to confirm the genetic determinism of body weight variability on a longer lifespan. However, the links between this ability to express a more or less homogeneous body weight and classical (i.e. physiological and/or behavioral) indicators of sensitivity to the environment have not yet been investigated. Therefore, the main aim of the study was to investigate, for the same batch of animals, whether physiological and behavioral phenotypes were correlated with phenotypic plasticity of body weight. The body weight and physiological parts of the study represent original experimental data. The behavioral part of the study is based on data previously published in Millot *et al*. [25] that was re-analyzed for the specific purpose of the present study.

## Materials and methods

### Ethical statement

PEIMA INRA facilities are authorized for animal experimentation under French regulation C29-277-02. The experiments were carried out in 2007–2008 in accordance with the Guidelines of the National Legislation on Animal Care of the French Ministry of Research (Decree N°2001–464, May 29, 2001). At this date, project authorization was not required. Experiments were conducted under the official licence of M. Dupont-Nivet (A29102), P. Prunet (07393) and M.L. Bégout (17–010).

### Biological material: Production of heterozygous isogenic lines

Experimental fish were produced and reared in the INRA experimental fish farm (PEIMA, Sizun, France). INRA homozygous isogenic lines, previously established by Quillet *et al*. [23] and maintained at PEIMA by single within-line pair mating, were used as breeders. Ten heterozygous isogenic lines were produced by mating several homozygous females from a single isogenic line (B57) with ten homozygous sex-reversed XX males from 10 other isogenic lines (A02, A03, A22, A36, AB1, AP2, B45, B61, N38 and R25). In order to obtain enough eggs, eggs collected from 24 females were used. To minimize bias due to maternal effects and to minimize the initial environmental variance, these females were chosen among 200 homozygous females from line B57 because they had spawned on the same day and had similar egg weight. Eggs were mixed and then divided into ten batches, each batch being fertilized by one of the 10 homozygous males. Using this mating scheme, the between-line differences were expected to be genetic ones only, brought about by the male pathway. It also avoided inbreeding effects likely to occur in homozygous lines. Previous results showed that performances of these heterozygous lines are in the range of those of conventional trout from the original population. To avoid confusion with the INRA homozygous isogenic lines, heterozygous lines will be named A02h, A03h etc.

**Fig 1:**
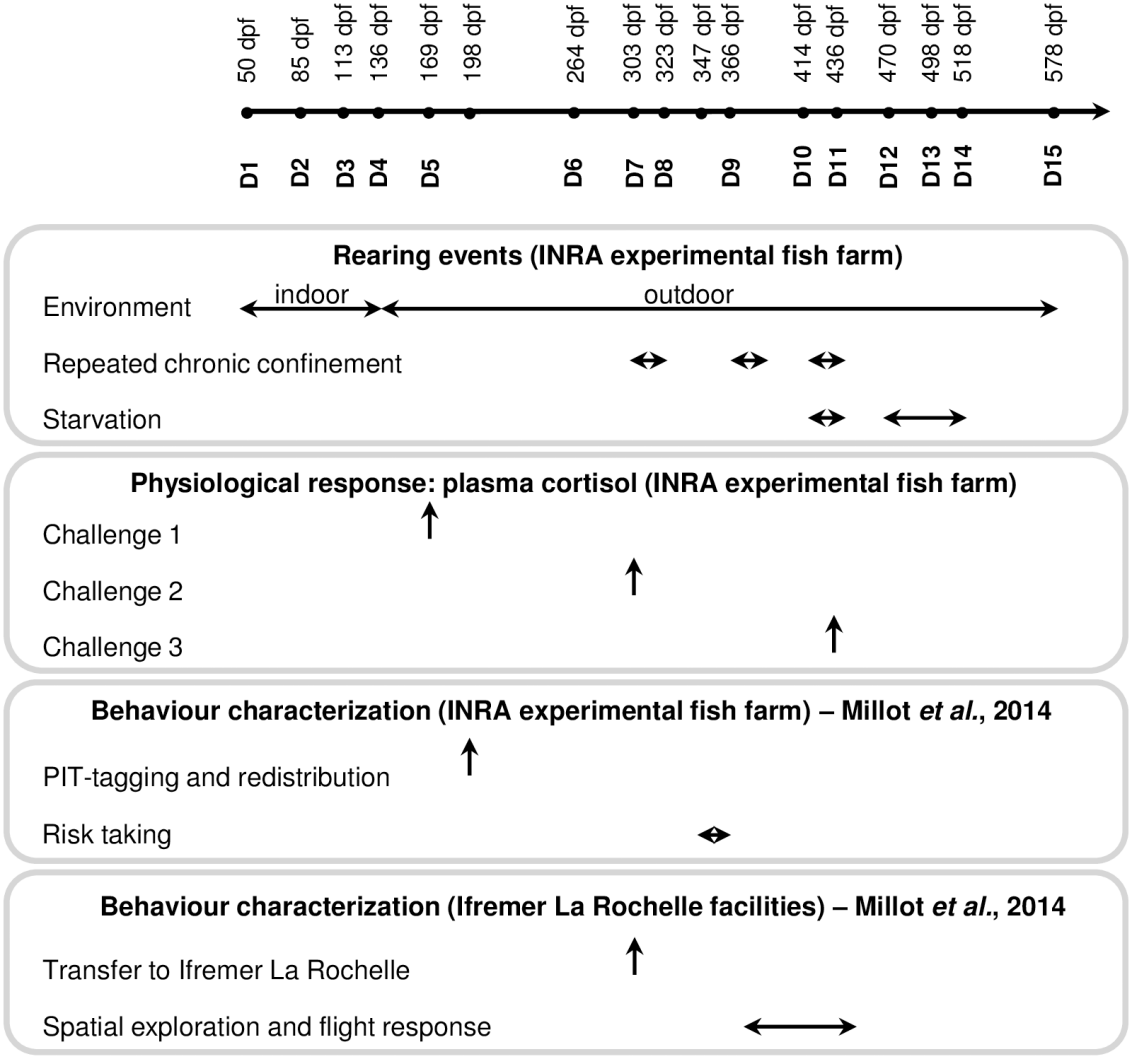
Experimental procedure. dpf: days post fertilization.

Fertilized eggs were incubated at 11.4°C. At eyed stage, 1500 eggs of each of the lines were distributed into three replicates (500 per replicate) for rearing in 0.25 m^3^ indoor tanks supplied with natural spring water (11.4°C). At 137 dpf (see Fig 1) fish from each tank were transferred to 1.8 m^3^ outdoor tanks supplied with river water (11.3–16°C). Each replicate was started with 400 fish, then it decreased by random elimination to keep low density in each tank (below 50 kg.m^-3^), until 100 fish remained at the end of the experiment at 578 dpf. Fish were fed *ad libitum* on a commercial diet (BioMar’s biostart range and Le Gouessant’s B-Mega range), with automatic distribution during the indoor phase and with self-feeders (Imetronic^®^, France) during the outdoor rearing phase.

During the outdoor rearing phase, repeated chronic confinement was performed from 305 dpf (after measure D7) to 435 dpf (until measure D11) and involved three cycles of confinement phase interspaced with a recovery phase (Table 1). Also, fish were deprived of food for 22 days between 414 dpf (D10) and 436 dpf (D11); for 48 days between 470 dpf (D12) and 518 dpf (D14) (Fig 1).

**Table 1:** Details of confinement challenges.

Challenge	Type	Date (dpf^a^)	Water level reduction	Mean density (kg.m^-3^)
Challenge 1	Acute confinement	169	25% (1.88 m^3^ down to 0.47 m^3^)	20.3 ± 3.3
Challenge 2	Acute confinement	302	17% (1.88 m^3^ down to 0.31 m^3^)	178.6 ± 19.4
Challenge 3	Confinement Cycle 1	305 to 323	25% (1.88 m^3^ down to 0.47 m^3^)	106.9 ± 12.7
	Recovery phase	324 to 367	N/A	N/A
	Confinement Cycle 2	368 to 379	33% (1.88 m^3^ down to 0.63 m^3^)	93.6 ± 13.6
	Recovery phase	380 to 415	N/A	N/A
	Confinement Cycle 3	416 to 435	28% (1.88 m^3^ down to 0.53 m^3^)	129.2 ± 23.0
	Acute stress	435	Netting the fish out of the water during a few seconds	

^a^dpf: days post fertilization

### Within-line body weight variability: A proxy of the line environmental sensitivity

Approximately 100 randomly sampled fish per replicate per line were weighed, from 50 dpf to 578 dpf, at a monthly interval. In total, there were 15 successive measurements for body weight (D1 to D15, Fig 1). In addition, total body length and Fulton’s condition factor (K = 100(BW/L^3^), where W is the body weight in grams and L the length in centimeters) were measured at D5 and D15. No measurement was taken during the summer period to avoid further stressing the fish experiencing water temperature higher than their optimum.

In order to examine within-line body weight variability, the body weight coefficient of variation (CV = (standard deviation / mean) * 100) was calculated by replicate, line and date. CV allows the comparison of variability between lines and is a meaningful parameter often used by breeders to assess the quality of rearing since high CVs are indicators of poor environmental conditions. Also, because there is no within-line genetic variability, the within-line (among fish within tanks) phenotypic variance (assessed here by CV body weight) is an estimator of the random environmental variance, i.e. the environmental sensitivity of the line genotype.

A general linear mixed model for longitudinal data (PROC MIXED in SAS, with REPEATED statement) was fitted to test the effect of the isogenic line on CV of body weight:

$${\text{CV}}_{\text{ijk}}{=\mu +\text{line}}_{\text{i}}{+\text{time}}_{\text{j}}{+\text{line}}_{\text{i}}{*\text{time}}_{\text{j}}{+\text{residual}}_{\text{ijk}}$$

where CV is the coefficient of variation for body weight in % for each replicate; μ is the overall mean; line, time and interaction line*time are fixed effects. This analysis was performed on two distinct rearing periods: indoor (D1 to D4; 10 lines * 4 dates * 3 replicates = 120 observations) and outdoor (D5 to D15; 10 lines * 11 dates * 3 replicates = 330 observations) (Fig 1). For the covariance structure describing the repeated measures design of the data, we have modeled several covariance structures, with homogeneous or heterogeneous variances. Based on the information criteria (AIC, AICC or BIC), no common model could be found for the two periods. Since the choice of the covariance structure did not have any impact on the estimation of the fixed effects (data not shown), we have chosen to use the same model for the two periods, the unstructured model. This is the most complex covariance structure, which estimates unique correlations for each pair of time points. Fisher’s least-square difference (LSD) test was performed using the LSMEANS statement to investigate pairwise differences among isogenic lines over each period. False discovery rate (FDR) procedure [26] was implemented to account for multiple testing.

### Physiological indicators of environmental sensitivity: Confinement challenges and cortisol assays

The line response to stressful environment was assessed by performing three confinement challenges associated with different stressor intensity: challenges 1, 2 and 3 (Fig 1). Challenges 1 and 2 were conducted at 169 dpf (average weight of 25 g) and 302 dpf (average weight of 259 g) respectively, and consisted in reducing the water level during one hour (Table 1). Challenge 3 was a repeated chronic confinement stress performed from 305 dpf during 130 days: fish were exposed to 3 periods of high rearing density interspaced by 2 recovery phases (Table 1 and Fig 1). At the end of the third period of high density (at 435 dpf, see Fig 1), fish were exposed or not to an acute stress consisting of netting the fish out of the water during a few seconds. Indeed, previous results showed that a single measurement of plasma cortisol concentration at the end of a high-density period is not a reliable measure to evaluate fish response to chronic stress [27]. Instead, it has been shown in salmonids that the application of an acute stressor helps to reveal a chronic stress situation [28,29].

For each challenge, the physiological response of the 10 isogenic lines was measured before and after confinement challenge through cortisol levels. At each sampling date, 10 fish per replicate per line were sampled before the confinement for basal cortisol levels and 10 separate fish after the confinement for post-stress cortisol levels. Fish were netted and euthanized immediately in water containing excess 2-phenoxyethanol (10 ml.l^-1^ water). Blood samples were collected from the caudal vein into a heparinized syringe and the fish were individually weighed. Blood samples were immediately placed on ice, then centrifuged at 4°C and the plasma transferred to new tubes that were immediately frozen at -80°C before transfer to -20°C until cortisol measurements. Individual plasma cortisol concentrations (ng.ml^-1^) were determined according to the radioimmunoassay procedure detailed in Aupérin *et al*. [30].

Basal and post-stress cortisol data of each confinement challenge were analyzed separately by a one-way ANOVA using PROC GLM in SAS, with the following statistical model:

$${\text{cortisol}}_{\text{ijk}}{=\mu +\text{line}}_{\text{i}}{+\text{replicate}}_{\text{j}}\left({\text{line}}_{\text{i}}\right){+\text{residual}}_{\text{ijk}}$$

where cortisol is the observed plasma cortisol level in ng.ml^-1^; μ is the overall mean per challenge; line is a fixed effect and replicate(line) is a random nested effect. To test for significant differences in cortisol level among isogenic lines, the F-test for the line effect was carried out by using the nested effect replicate(line) as the error term. When significant differences among isogenic lines were detected, Fisher’s LSD test was performed using the LSMEANS statement of PROC GLM.

### Behavioral indicators of environmental sensitivity: Spatial exploration, flight response and risk taking

Lines were also evaluated for behavioral traits. Detailed experimental set-up for these tests and results were published in Millot *et al*. [25]. For the purpose of the present study, we removed redundant variables (i.e. highly correlated) in order to identify a subset of variables most representative of the different facets of the lines’ behavioral features. It is important to note that fish used for the behavioral characterization were part of the same experiment studying body weight and physiological response measurements. They were reared in the same tanks until 198 dpf, when they were PIT-tagged and redistributed for the behavioral assessment (Fig 1).

Firstly, risk-taking behavior was characterized at INRA experimental fish farm between 347 and 363 dpf for the ten isogenic lines mixed in three replicates of 500 fish (50 PIT-tagged fish per line x 10 lines). Full description of the test is given in Millot *et al*. [31]. Briefly, the experimental tank was separated into two equal zones (safe (shade) vs. risky (light)) by an opaque divider with a circular opening equipped with a PIT-tag detection antenna. At the beginning of the experiment, all fish were gathered in the safe zone, before the opening of the opaque divider, allowing the fish to move from the safe to the risky zone. The two measurements associated with risk-taking behavior chosen were: the average percentage of time spent by each fish in the risky zone (RT_%_time_spent) and the average number of passages through the opening per fish (RT_ntpass) (Table 2).

**Table 2:** Description and abbreviation of the behavioral traits (from Millot *et al*. [25]) used in this study.

Experiment	Description	Abbreviation
Risk taking (RT)	Average percentage of time spent by each fish in the risky zone	RT_%_time_spent
	Average number of passages through the opening per fish	RT_ntpass
Spatial exploration (SPE)	Average proportion of time spent by a fish in each zone during Sequence 1 (before the stimulus fall). Z1 (stimulation zone) to Z4 (furthest away from stimulation zone).	SPE_Seq1_Z1
		SPE_Seq1_Z2
		SPE_Seq1_Z3
		SPE_Seq1_Z4
	Average proportion of time spent by a fish in each zone during Sequences 2 and 3 (after the stimulus fall). Z1 (stimulation zone) to Z4 (furthest away from stimulation zone).	SPE_Avg23_Z1
		SPE_Avg23_Z2
		SPE_Avg23_Z3
		SPE_Avg23_Z4
Flight response (FR)	Difference in distance travelled between Sequences 2 and 1, in response to the stimulus fall (in m)	FR_Dist_diff21
	Average distance travelled by each fish during Sequence 2 plus Sequence 3 (after stimulus fall; in m)	FR_Dist_Seq23

Secondly, 140 tagged fish were transferred from PEIMA to Ifremer La Rochelle facilities at 304 dpf (mean weight: 157.3 ± 50.2 g) in order to assess spatial exploration ability after isolation and flight response to a stimulus. The stimulus is the sudden fall of an object in the experimental tank. 7 out of the 10 heterozygous isogenic lines (all lines except A22h, A36h and N38h; 10 fish per line) were characterized between 374 and 444 dpf (full test description is given in Millot *et al*. [32]). Briefly, a single fish at a time was moved from its maintenance tank (400 l) to the experimental tank (400 l). After 2h of acclimatization, the activity and position of the fish in the experimental tank was recorded during three 20-minute sequences: sequence 1 (before the stimulus fall), sequence 2 (just after the stimulus fall) and sequence 3 (40 min after the stimulus fall). Analysis of video recordings allowed the separation of the tank into four virtual zones of equal surface: Z1 (stimulation zone) to Z4 (the furthest away from the stimulation zone). For the present study, eight variables were selected to describe spatial exploration behavior: the average proportion of time spent by a fish in each zone before (SPE_Seq1_Z1 to Z4) and after the stimulus fall (SPE_Avg23_Z1 to Z4). Two variables were selected to describe flight response: the difference in distance travelled between Sequences 2 and 1 (FR_Dist_diff21) and the average distance travelled by each fish after the stimulus fall (FR_Dist_Seq23) (Table 2).

### Correlations between average CV of body weight, physiological and behavioral traits

In order to investigate the links between within-line body weight variability and classical (physiological and/or behavioral) indicators of environmental sensitivity, Pearson correlation coefficients between average CV of body weight (indoor and outdoor), average body weight (indoor and outdoor), condition factor, cortisol levels and behavioral traits were obtained using PROC CORR in SAS. False discovery rate (FDR) procedure [26] was implemented to account for multiple testing. Also, in order to summarize this multivariate dataset and to group the isogenic lines based on the variables (average CV of body weight, average body weight, condition factor, post-stress cortisol levels and the behavioral traits) characteristics, a Principal Components Analysis (PCA) was performed using the FactoMineR package in R [33]. The rainbow trout isogenic lines and the variables were plotted on a 2D plot.

## Results

At the end of the experiment (578 dpf), fish weighed on average 815 g, with a generally low mortality, involving a few fish per month. Two periods were however associated with higher mortality: at the very beginning (until 50 dpf), with mortality up to 5.4% and during the summer months (169–207 dpf), associated with poor water quality and occurrence of pathogens. The 3 lines mostly affected by the summer peak of mortality were R25h (28.5% mortality overall 3 replicates), N38h (18.8%) and A36h (15.9%), and to a lesser extent A03h (9.7%). In contrast, lines A22h and AB1h were the least affected, with 0.6% and 1.4% mortality over the same period.

### Coefficient of variation (CV) of body weight

**Fig 2:**
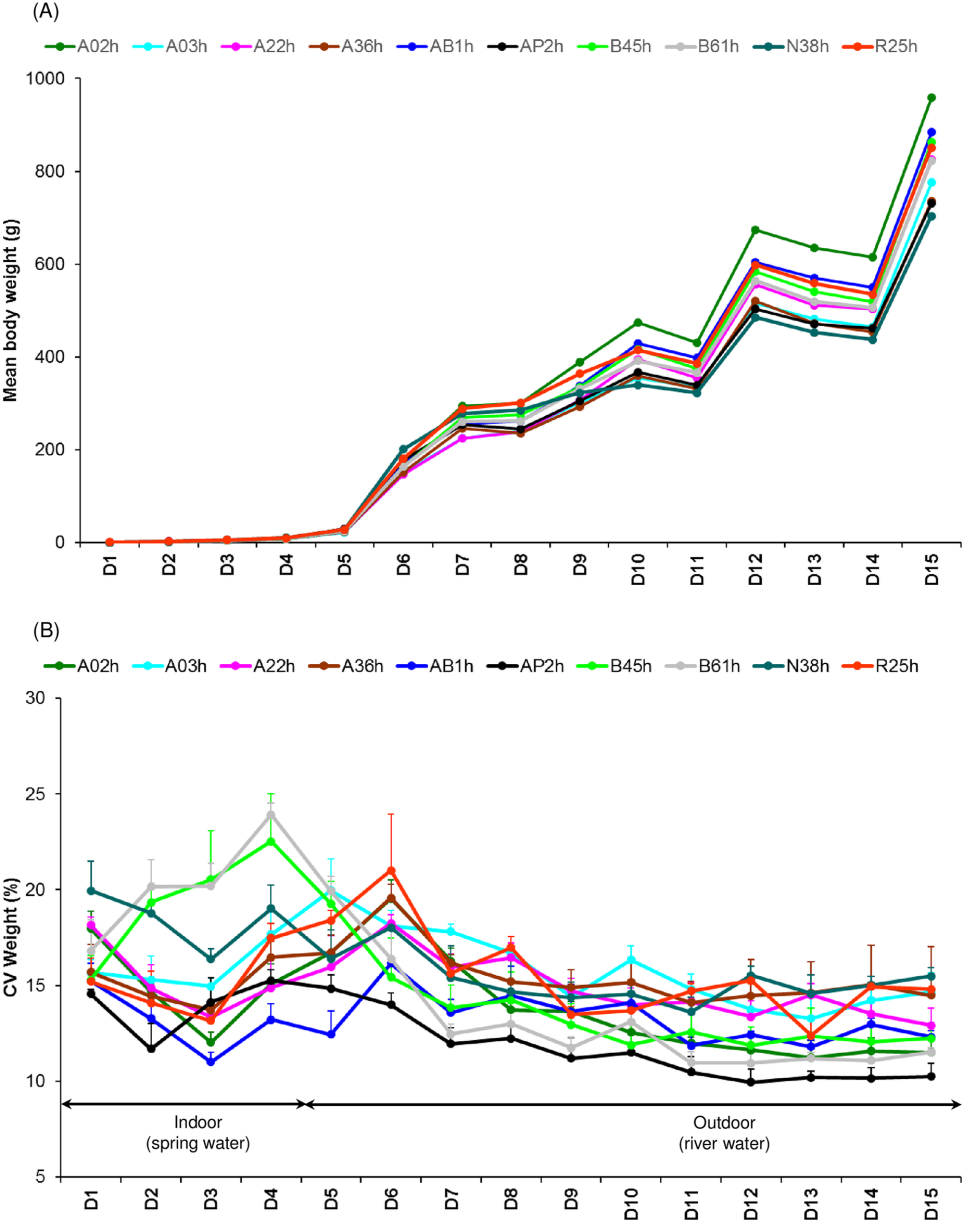
The changes of mean body weights and coefficient of variation (CV) for body weight (mean ± SEM, in %) over the period of experiment. Each dot represents the mean of three replicates. SEM: standard error of the mean.

The changes of mean body weights and CV body weight over the course of the experiment are represented in Fig 2. Overall, CV body weight ranged from 9.94 to 23.92%. Some general features can be highlighted: i) An initial re-ranking of the lines in the period D1-D2; ii) A peak in CV body weight for the lines B45h and B61h in the period D3-D5; iii) Lower variability in CV body weight among the lines in the period D9-D15, with almost no re-ranking.

**Fig 3:**
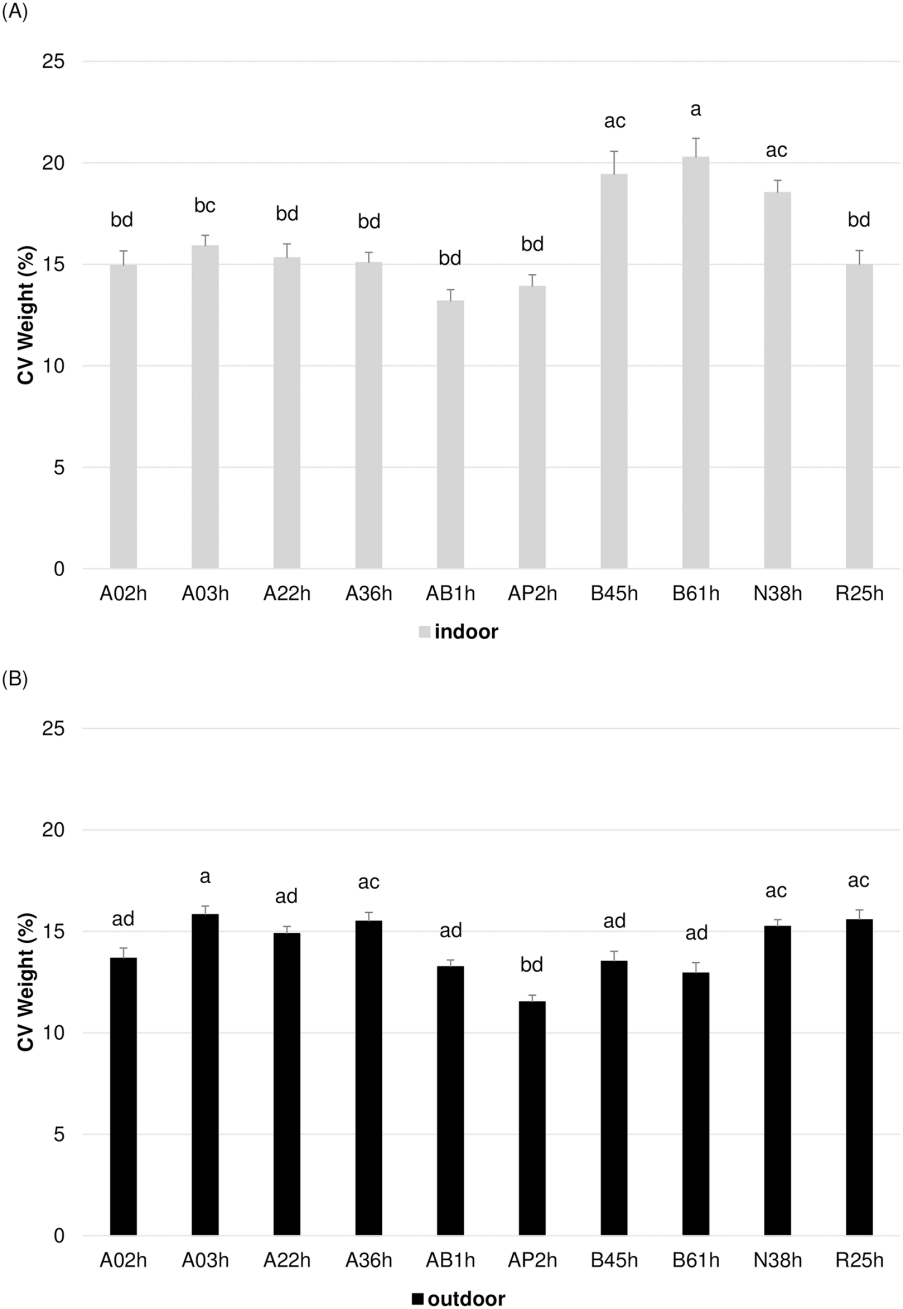
Mean CV body weight ± SEM for each period: a) indoor and b) outdoor. Means with a different superscript are significantly different (P<0.05).

Longitudinal analyses with PROC MIXED in SAS using the REPEATED statement were done on 2 periods: indoor (D1 toD4) and outdoor (D5 to D15) (Fig 2). For each period, there were significant line, time and line*time effects (Table 3). The longitudinal analyses showed that lines B45h (19.42 ± 1.15%), B61h (20.27 ± 0.93%) and N38h (18.53 ± 0.60%) were significantly more variable than the other 7 lines (14.76 ± 0.34%) indoor (Fig 3a; S1 Table). Outdoor, lines A03h (15.82 ± 0.43%), A36h (15.49 ± 0.44%), N38h (15.24 ± 0.34%) and R25h (15.57 ± 0.48%) were the most variable, while AP2h was the least variable (11.52 ± 0.34%) (Fig 3b; S1 Table).

**Table 3:** Repeated analyses CV body weight for unstructured (un) covariance structure.

Date	Effect					
	line		time		line*time	
	F value (Num DF^b^, Den DF^b^)	P^c^	F value(Num DF^b^, Den DF^b^)	P^c^	F value(Num DF^b^, Den DF^b^)	P^c^
Indoor^a^	10.34(9, 20)	<0.001 ***	18.83(3, 20)	<0.001 ***	2.63(27, 20)	0.014 *
Outdoor^a^	4.30(9, 20)	0.003 **	32.88(10, 20)	<0.001 ***	5.43(90, 20)	<0.001 ***

^a^ Indoor: D1 to D4; Outdoor: D5 to D15

^b^ Num DF: numerator degrees of freedom; Den DF: denominator degrees of freedom

^c^ * P<0.05; ** P<0.01; *** P<0.001

### Cortisol level after confinement challenge

Cortisol levels before and after confinement are shown in S1 Fig, for each of the three challenges. Generally, post-stress cortisol levels were much smaller for Challenge 1 (26.5 ± 1.2 ng.ml^-1^) than for Challenge 2 (74.4 ± 4.3 ng.ml^-1^) and Challenge 3 (97.4 ± 8.9 ng.ml^-1^).

For Challenge 1, in the control and the stressed groups, there were no significant differences in cortisol level among the lines (F_9,282_ = 0.40, P = 0.919; F_9,282_ = 1.09, P = 0.411 respectively). However, line R25h exhibited unusually high variability in post-stress cortisol levels, up to 34 ng.ml^-1^ difference between the 3 replicates compared with the other lines (inter-replicate variability ranging from 1 to 12 ng.ml^-1^). After removing the line R25h, there were significant differences in post-stress cortisol level among the lines (F_8,255_ = 3.19, P = 0.019): cortisol level was significantly higher for line B45h (34.7 ± 5.2 ng.ml^-1^) than lines A02h (22.5 ± 2.5 ng.ml^-1^), A22h (22.9 ± 2.5 ng.ml^-1^), AB1h (22.8 ± 2.8 ng.ml^-1^) and N38h (24.1 ± 2.9 ng.ml^-1^) (S1 Fig).

For Challenge 2, there were no significant differences in cortisol level among the lines in the control group (F_9,290_ = 2.27, P = 0.061). However, after confinement, there were significant differences among the lines (F_9,290_ = 5.48, P<0.001): lines A36h (95.1 ± 3.0 ng.ml^-1^), N38h (87.4 ± 3.4 ng.ml^-1^) and R25h (97.0 ± 4.5 ng.ml^-1^) showed a higher cortisol level than the other 7 lines (66.4 ± 1.4 ng.ml^-1^) (S1 Fig).

For Challenge 3, there were significant differences in cortisol level among lines in the control (F_9,290_ = 5.83, P<0.001) and stressed (F_9,290_ = 14.96, P<0.001) groups. In the control group, lines A22h (39.6 ± 3.0 ng.ml^-1^) and AB1h (54.6 ± 4.4 ng.ml^-1^) exhibited a significantly higher cortisol level than the other lines (18.5 ± 2.5 ng.ml^-1^), except AP2h (32.5 ± 2.4 ng.ml^-1^). In the stressed group, line AB1h had the highest cortisol level (134.4 ± 6.0 ng.ml^-1^) (S1 Fig).

### Behavior data

Detailed results and statistical analyses have been published in Millot *et al*. [25].

For the risk taking experiment, data was available for the 10 isogenic lines. Significant behavioral differences between lines were observed, with lines A03h, B61h and B45h exhibiting higher risk taking behavior than lines A02h, A22h and AP2h (S2 Table). For the spatial exploration and flight response experiment, data was available for 7 isogenic lines. There were significant differences among lines in spatial exploration behavior in a new environment: line A03h showed a low level of swimming activity (54.3 ± 20.4 m), while line A02h showed homogeneous swimming and high spatial exploration behavior (367.3 ± 63.0) (S3 Table). Also, although all lines showed a decrease in swimming activity after the stimulus fall (i.e. freezing behavior), lines B45h and B61h showed the highest decrease (-85.0 ± 19.9 m and -82.4 ± 20.5 m respectively) and line A02h the lowest (-10.6 ± 26.0 m) (S3 Table).

### Integrative assessment of performances: Correlations between average CV of body weight, cortisol levels and behavioral traits

**Fig 4:**
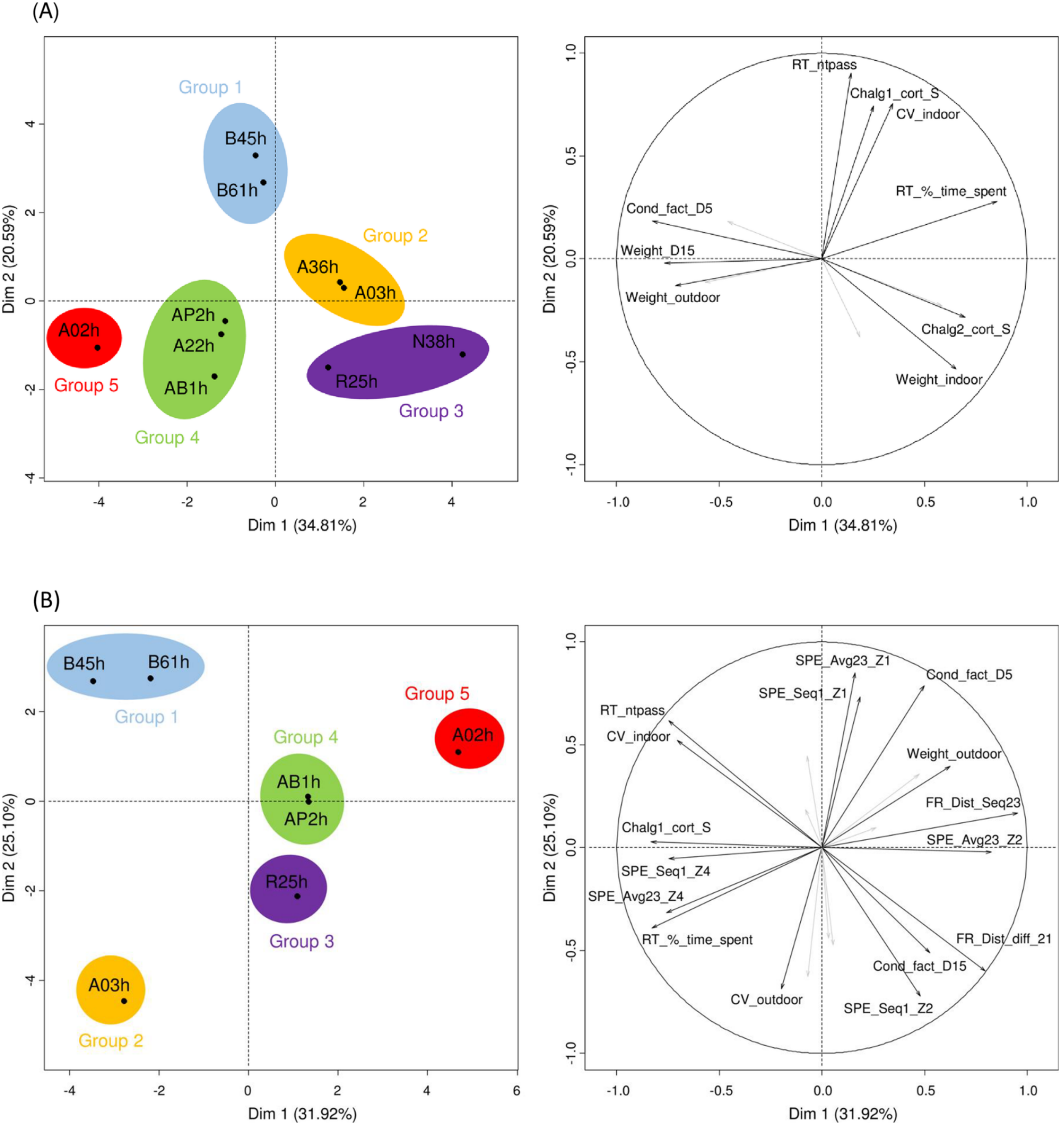
Principal component analysis. (A) 10 lines for CV body weight, average body weight, condition factor, risk taking behavior and cortisol levels after confinement challenges; (B) 7 lines for CV body weight, average body weight, condition factor, risk taking behavior, spatial exploration and flight response traits, and cortisol levels after confinement challenges. Variables with cos2 < 0.5 are shown in grey with no labels (the cos2 values are used to estimate the quality of the representation for variables on the factor map).

PCA analyses were performed, including average CV of body weight during 2 periods (indoor and outdoor), average body weight, condition factor, post-stress cortisol levels for the 3 confinement challenges and the 12 behavioral traits listed in Table 2. The first 2 axes explained 55% of the variance for the analysis including the 10 isogenic lines (Fig 4a, behavior data including risk taking only), and 57% of the variance for the analysis including the 7 isogenic lines (Fig 4b, behavior data including risk taking but also spatial exploration and flight response).

Analysis performed with the 10 isogenic lines revealed several groupings (Fig 4A). Group 1 included lines B45h and B61h, associated with high CV body weight (indoor), high risk taking behavior (high RT_ntpass) and high cortisol level after Challenge 1. Group 2 included lines A03h and A36h, associated with high CV body weight (outdoor), low average body weight (outdoor) and high risk taking behavior (high RT_%_time_spent). Group 3 included lines N38h and R25h, associated with high CV body weight (outdoor), high average body weight (indoor), low condition factor at the end of the indoor period (Cond_fact_D5), low risk taking behavior (low RT_ntpass) and high cortisol level after Challenge 2 but low cortisol level after Challenge 3. Group 4 included lines A22h, AB1h and AP2h, associated with low CV body weight (indoor), low risk taking behavior (low RT_ntpass and RT_%_time_spent), low cortisol levels after Challenges 1 and 2 but high cortisol level after Challenge 3. Group 5 included line A02h, associated with low CV body weight (indoor and outdoor), high average body weight (outdoor), high condition factor (at dates D5 and D15), low risk taking behavior (low RT_ntpass and RT_%_time_spent) and low cortisol levels after the 3 confinement challenges. The direction of the arrows on the graph of variables corroborates the correlations listed in S4 Table: strong positive correlation between CV_indoor and RT_ntpass (r = 0.70, P = 0.023); positive correlation between CV_outdoor and Chalg2_cort_S (r = 0.60, P = 0.069); negative correlations between CV_outdoor and Chalg3_cort_S or Cond_fact_D5 (r = -0.62, P = 0.054; r = -0.62, P = 0.057 respectively).

Analysis performed with the 7 isogenic lines confirmed the main groupings above (Fig 4B). Lines B45h and B61h (Group 1) were associated with low flight response (low FR_Dist_diff_21 ranging -82 to -85 m: freezing behavior) and spent on average more time in zone 1 than other lines (high SPE_Seq1_Z1 and SPE_Avg23_Z1). Line A03h (Group 2) spent on average less time in zone 1 (low SPE_Seq1_Z1 and SPE_Avg23_Z1) and more time in zone 4 (high SPE_Seq1_Z4 and SPE_Avg23_Z4). Lines R25 (Group 3), AB1h and AP2h (Group 4), and A02h (Group 5) were associated with high flight response (high FR_Dist_diff_21 ranging -10 to -30 m), high spatial exploration ability (high SPE_Dist_Seq23) and spent on average less time in zone 4 (low SPE_Seq1_Z4 and SPE_Avg23_Z4). The direction of the arrows on the graph of variables corroborates the correlations listed in S5 Table: strong negative correlations between CV_indoor and FR_Dist_diff_21 (r = -0.88, P = 0.009); CV_outdoor and Cond_fact_D5 (r = -0.83, P = 0.020); CV_outdoor and Chalg3_cort_S (r = -0.78, P = 0.040); negative correlation between CV_indoor and SPE_Avg23_Z2 (r = -0.71, P = 0.077); strong positive correlation between CV_indoor and RT_ntpass (r = 0.92, P = 0.005).

## Discussion

To cope with environmental conditions, organisms can put in place a complex set of physiological and / or behavioral responses. Hence, it is important to understand how organisms adapt to fluctuating environmental conditions or respond to environmental stress. In this context, the present study aimed at investigating the genetic variability of sensitivity to environmental fluctuations, assessed here by coefficient of variation of body weight, and its correlations to physiological and behavioral traits.

### Are some genotypes more sensitive to their environment than others?

While working in natural populations, it can be difficult to disentangle genetics from environmental factors involved in phenotypic plasticity. To answer this question, it is therefore easier and more powerful to use simpler biological models in a controlled setting where environmental factors can be controlled and/or monitored. Moreover, in animal breeding-oriented approaches, the genetic determinism of environmental sensitivity is usually assessed by complex statistical models [21,34] relying on large family structures. For example, genetics of environmental variation in rainbow trout has been studied by using a variance model with the log-transformed squared residual values of a bivariate animal model as input data [35]. One of the strengths of the present study is to rely on original biological material, the rainbow trout isogenic lines [23], which allowed us to disentangle the genetic and environmental parts of the phenotypic variance and to directly investigate the genetic determinism of environmental sensitivity by looking at the within-line (among fish within tanks) phenotypic variance. We focused on phenotypic variance of body mass (100 animals per line per replicate measured) since, for other traits, the number of animals (10 per line per replicate for physiological traits; 10 to 50 per line per replicate for behavioral traits) precludes accurate estimation of residual environmental variances. This study unambiguously demonstrated the existence of genetic determinism of environmental sensitivity, with some lines (B45h and B61h) being particularly sensitive to environmental fluctuations (particularly during indoor period) and others (e.g. AP2h) rather insensitive. Compared with a previous study using the same biological material (heterozygous isogenic lines but with different genetic backgrounds, [24]), the present study was based on a longer time period (18 months), with weight measurements taken every month, which allowed us to finely monitor the changes of environmental sensitivity over time. The existence of genetic determinism of phenotypic plasticity for body weight is quite relevant in the context of the ability of the fish to adapt to fluctuating environmental conditions. It should be noted that the analysis assumed that the environment was common among the lines. However, this may not be the case. The lines could be experiencing uncontrolled differences in their environment (e.g. social interactions) that might induce differing levels of inter-individual phenotypic variation.

### Which factors influence environmental sensitivity?

Besides the genetic control highlighted by the existence of genetic determinism of environmental sensitivity, changes in the environment seemed to be a key determinant factor of phenotypic fluctuation between and within lines. Indeed, two lines (B45h and B61h) exhibiting a high coefficient of variation (CV) indoors (period D3-D4) were then re-ranked and showed a low CV (i.e. low environmental sensitivity) after transfer to outdoor rearing tanks (period D7-D15). It is important to note that peaks of variability were not due to a single replicate exhibiting higher variability than the other replicates. The transfer to outdoor tanks was associated with changes in water quality, tank depth, lighting and feeding management: temperature variations were not buffered by the building, lighting changes during the year and water quality was not stable since river water was used. Moreover, fish were fed with mechanical distribution indoors, but with self-feeders outdoors. On the whole, the environment was much less controlled outdoors and, although an increase of body weight CV could be expected, our observations show the opposite.

However, in our experiment, effects of rearing place (indoor vs. outdoor) were confounded with age. There were many re-rankings of the lines during the very early phases (period D1-D2, indoor). From period D7 onwards (outdoor), there was less amplitude in the variation of the CV of the 10 isogenic lines, with all lines within 5% CV from each other (Fig 2). This is even more surprising because several rearing events were expected to impact body weight variability outdoor i.e. repeated chronic confinement and starvation (Fig 1). Two hypotheses can be put forward to explain why the main differences in phenotypic plasticity between lines occurred during the early phases. The first hypothesis is size-correlated bias in the coefficient of variation. Because of the overall reduction of growth potential with size, the expected relative weight gain between two successive measurements decreases as fish become larger. Therefore, the relative changes in CV values are expected to diminish with time, limiting the chance to observe re-ranking among lines. The second hypothesis is that young fish are more sensitive to environmental fluctuations than adults [36]. However, no experimental data in the present study substantiate this hypothesis, so our conclusions should be confirmed in further experiments.

Finally, re-ranking of the lines occurred at D6, with lines R25h, A36h, A02h, A22h and AB1h exhibiting a peak of CV body weight (Fig 2). This peak of phenotypic variability followed the summer period associated with poor water quality and mortality caused by the pathogen *Flavobacterium psychrophilum*. However, it is interesting that lines differed in their response to this summer mortality period. Indeed, among the lines exhibiting a peak of CV body weight at D6, two were among the most affected by the summer peak of mortality (R25h and A36h with 28.5% and 18.8% mortality respectively), and two among the least affected (A22h and AB1h with only 0.6% and 1.4% mortality). Therefore, we cannot rule out that poor water quality and occurrence of pathogens over the summer period played a role in the re-ranking of the lines, but it is unlikely to be the predominant factor.

### Are the different indicators of environmental sensitivity consistent?

Generally, physiological [15] and behavioral [16] indicators are used to assess adaptation potential of a fish and hence its ability to cope with environmental stressors. The strength and novelty of the present study is to investigate the link between environmental sensitivity assessed globally (by within-line phenotypic variance: average coefficient of variation for body weight) and more targeted physiological and behavioral indicators. Trying to find the phenotypes that best characterize environmental sensitivity is crucial in the current socio-economic context of sustainable aquaculture under the global change. PCA and correlations results highlighted two extreme cases: i) “Sensitive” genotypes, sensitive to new events or environmental changes; ii) “Non sensitive” genotypes, not sensitive to environmental changes. This dichotomy between sensitive vs. non sensitive genotypes can be paralleled to the two opposite coping styles, proactive vs. reactive [37,38]. Coping style encompasses both behavioral and physiological (neuroendocrine and metabolic) responses of an organism to cope with stress [37]. It has been established that animals can show two opposite stress-coping strategies, proactive (high risk taking behavior, high flight response and low cortisol responsiveness) or reactive (low risk taking behavior, low flight response and high cortisol responsiveness) [37], also well recognized in fish (reviewed in [38,39]). However, in the present study, we prefer to use the terms sensitive / not sensitive because, as discussed below, the cortisol data are not in agreement with the coping styles theory.

An interesting finding of this study is that CV for body weight, a trait expected to be integrative of the whole life history of a fish, could be a good indicator of a genotype’s sensitivity to its environment and be linked to behavioral indicators (risk taking RT and flight response FR). Indeed, a significant correlation was found between CV body weight indoors and RT_ntpass (r = 0.70, P = 0.023 with 10 lines; r = 0.92, P = 0.005 with 7 lines) and FR_Dist_diff_21 (r = -0.88, P = 0.009). It is noteworthy that this relationship is only valid indoors, at the time when trout lines are the most divergent in terms of their response to the environment.

In contrast to behavioral indicators, cortisol responses to confinement challenges observed in this study are more difficult to interpret. Indeed, there was no consistent correlation between environmental sensitivity assessed by CV body weight and physiological indicators (post-stress cortisol levels). A significant correlation was found between CV_outdoor and Chalg3_cort_S (r = -0.78, P = 0.040) when considering 7 lines, but this correlation was not significant (r = -0.62, P = 0.054) with 10 lines. Also, physiological indicators were only poorly correlated with behavioral traits. This is surprising because plasma cortisol response after stress has been used as a selection criterion to select for high (HR) and low (LR) responding rainbow trout lines [40] and those lines were shown to differ in their behavioral responses [41–44].

Further studies will be needed to have a complete characterization of the coping style of our trout lines. The relationship between environmental sensitivity (average CV of body weight) and behavioral indicators should be addressed in a wider range of environmental stressors (type, severity and predictability), to investigate whether the relationship shown in this study stands or is context-dependent. Also, other physiological functions indicators (e.g. respiration, immune response…) should be considered in such studies, for a more complete characterization of the physiological response to stress.

In conclusion, the present study showed a certain consistency between average CV of body weight and some physiological and behavioral indicators of environmental sensitivity and confirms its potential to be included in environmental sensitivity assessment. Indeed, as the relationship between types of indicators is likely to depend on the nature and intensity of the stressor, the joint use of several types of indicators should help to investigate the biological complexity of environmental sensitivity. The results of this study have implications in the context of establishing efficient and sustainable aquaculture production systems where improving animal welfare and environmental sensitivity is becoming a priority. However, environmental sensitivity is a multi-facet trait, difficult to apprehend. Therefore, in order to include environmental sensitivity into breeding schemes, future research should include a diverse range of stressors and phenotypes to find the traits that best characterize environmental sensitivity.

## Supporting information

S1 Fig Cortisol levels before (control) and after (stressed) confinement, for three distinct challenges (mean ± SEM, in ng/ml). In Challenge 1, line R25 was removed from the statistical analysis because it exhibited unusually high variability in cortisol levels, up to 34 ng.ml^-1^ difference between the 3 replicates compared with the other lines (inter-replicate variability ranging 1 to 12 ng.ml^-1^). SEM: standard error of the mean.
(TIF)

S1 Table Results of the Fisher’s least-square difference (LSD) test (LSMEANS statement in SAS) for each of the two periods (indoor and outdoor). (DOCX)

S2 Table Risk taking behaviour data (mean ± SEM) for 10 rainbow trout isogenic lines. (DOCX)

S3 Table Flight response and spatial exploration data (mean ± SEM) for 7 rainbow trout isogenic lines. (DOCX)

S4 Table Correlations between line means of CV body weight, behavioural traits (risk taking) and cortisol levels for 10 isogenic lines. (DOCX)

S5 Table Correlations between line means of CV body weight, behavioural traits (spatial exploration SPE, flight response FR, risk taking RT) and cortisol levels for 7 isogenic lines. (DOCX)
