# Supplementary figures and images for: Genetic variability of environmental sensitivity revealed by phenotypic variation in body weight and (its) correlations to physiological and behavioral traits
Source: PLoS One. 2017 Dec 18;12(12):e0189943. doi: 10.1371/journal.pone.0189943 (PMC5734726; doi:10.1371/journal.pone.0189943)

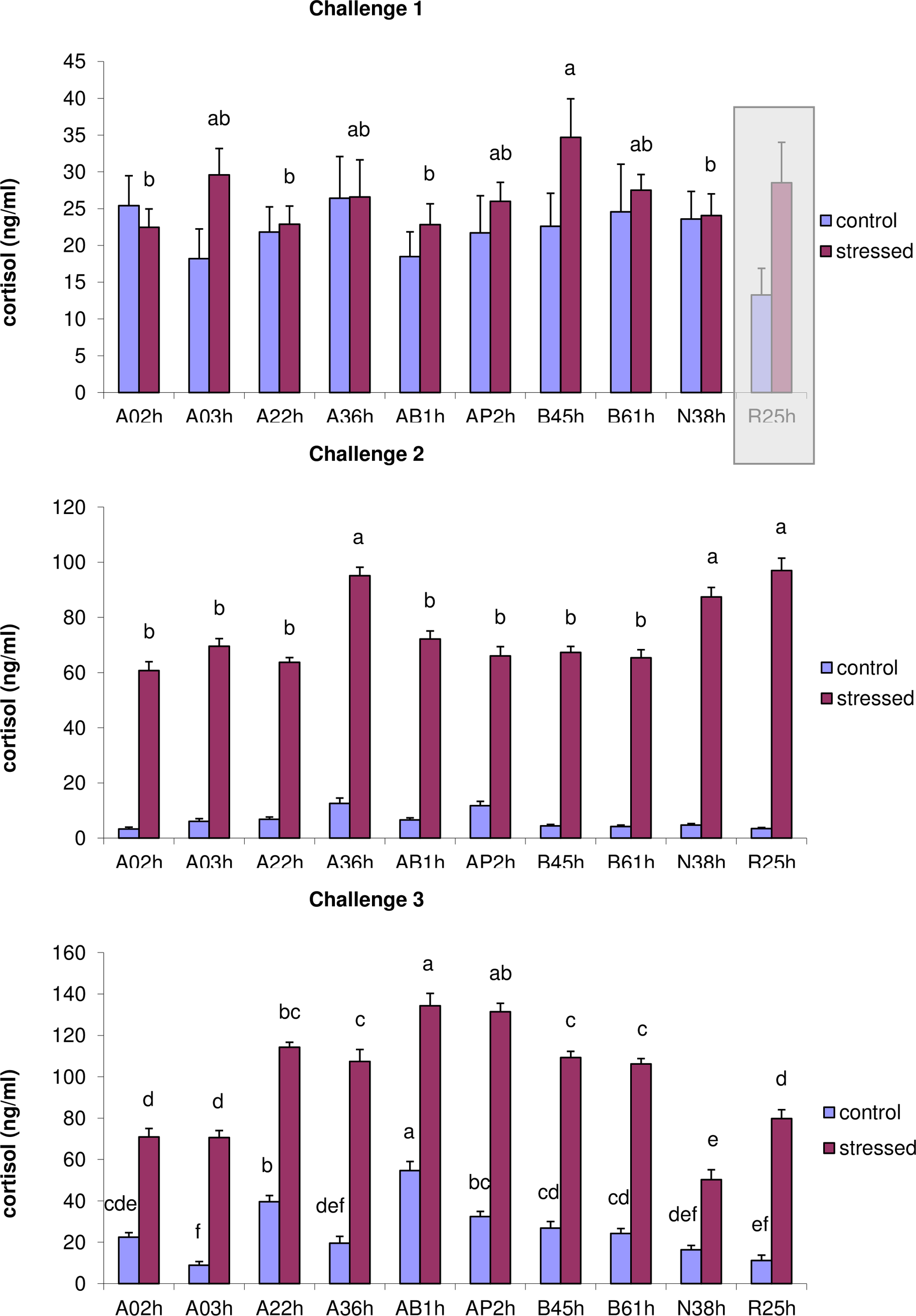

Supplement: S1 Fig — In Challenge 1, line R25 was removed from the statistical analysis because it exhibited unusually high variability in cortisol levels, up to 34 ng.ml-1 difference between the 3 replicates compared with the other lines (inter-replicate variability ranging 1 to 12 ng.ml-1). SEM: standard error of the mean. (TIF) [file pone.0189943.s001.tif]
