# Supplementary material for: Genetic variability of environmental sensitivity revealed by phenotypic variation in body weight and (its) correlations to physiological and behavioral traits
Source: PLoS One. 2017 Dec 18;12(12):e0189943. doi: 10.1371/journal.pone.0189943 (PMC5734726; doi:10.1371/journal.pone.0189943)
Supplement: S1 Table — (DOCX) [file pone.0189943.s002.docx]

**S1 Table Results of the Fisher’s least-square difference (LSD) test (LSMEANS statement in SAS) for each of the two periods (indoor and outdoor)**. In bold, P<0.011 (critical value for 45 tests for B-Y method Benjamini and Yekutieli 2001 to account for multiple testing).

| Comparison | DF | Indoor (D1 to D4) | | | Outdoor (D5 to D15) | | |
| --- | --- | --- | --- | --- | --- | --- | --- |
|  |  | Contrast | t-test value | P | Contrast | t-test value | P |
| A02-A03 | 20 | -0.97 | -0.91 | 0.372 | -2.15 | -2.22 | 0.038 |
| A02-A22 | 20 | -0.37 | -0.35 | 0.730 | -1.22 | -1.26 | 0.221 |
| A02-A36 | 20 | -0.14 | -0.14 | 0.893 | -1.83 | -1.89 | 0.074 |
| A02-AB1 | 20 | 1.75 | 1.66 | 0.113 | 0.42 | 0.43 | 0.670 |
| A02-AP2 | 20 | 1.03 | 0.98 | 0.339 | 2.15 | 2.22 | 0.038 |
| A02-B45 | 20 | **-4.49** | **-4.25** | **<0.001** | 0.14 | 0.15 | 0.884 |
| A02-B61 | 20 | **-5.33** | **-5.04** | **<0.001** | 0.73 | 0.75 | 0.460 |
| A02-N38 | 20 | **-3.59** | **-3.40** | **0.003** | -1.57 | -1.63 | 0.120 |
| A02-R25 | 20 | -0.04 | -0.04 | 0.970 | -1.90 | -1.97 | 0.063 |
| A03-A22 | 20 | 0.60 | 0.56 | 0.578 | 0.93 | 0.96 | 0.347 |
| A03-A36 | 20 | 0.82 | 0.78 | 0.445 | 0.33 | 0.34 | 0.739 |
| A03-AB1 | 20 | 2.72 | 2.57 | 0.018 | 2.57 | 2.66 | 0.015 |
| A03-AP2 | 20 | 2.00 | 1.89 | 0.073 | **4.30** | **4.44** | **<0.001** |
| A03-B45 | 20 | **-3.52** | **-3.33** | **0.003** | 2.30 | 2.37 | 0.028 |
| A03-B61 | 20 | **-4.36** | **-4.13** | **<0.001** | **2.88** | **2.98** | **0.007** |
| A03-N38 | 20 | -2.63 | -2.49 | 0.022 | 0.58 | 0.60 | 0.556 |
| A03-R25 | 20 | 0.92 | 0.88 | 0.392 | 0.25 | 0.26 | 0.799 |
| A22-A36 | 20 | 0.23 | 0.21 | 0.833 | -0.60 | -0.62 | 0.539 |
| A22-AB1 | 20 | 2.12 | 2.01 | 0.058 | 1.64 | 1.70 | 0.106 |
| A22-AP2 | 20 | 1.40 | 1.33 | 0.199 | **3.37** | **3.48** | **0.002** |
| A22-B45 | 20 | **-4.12** | **-3.90** | **<0.001** | 1.37 | 1.41 | 0.174 |
| A22-B61 | 20 | **-4.96** | **-4.69** | **<0.001** | 1.95 | 2.02 | 0.057 |
| A22-N38 | 20 | **-3.23** | **-3.05** | **0.006** | -0.35 | -0.36 | 0.720 |
| A22-R25 | 20 | 0.33 | 0.31 | 0.759 | -0.68 | -0.70 | 0.489 |
| A36-AB1 | 20 | 1.90 | 1.80 | 0.088 | 2.25 | 2.32 | 0.031 |
| A36-AP2 | 20 | 1.18 | 1.11 | 0.278 | **3.97** | **4.10** | **<0.001** |
| A36-B45 | 20 | **-4.34** | **-4.11** | **<0.001** | 1.97 | 2.03 | 0.055 |
| A36-B61 | 20 | **-5.18** | **-4.91** | **<0.001** | 2.56 | 2.64 | 0.016 |
| A36-N38 | 20 | **-3.45** | **-3.27** | **0.004** | 0.25 | 0.26 | 0.797 |
| A36-R25 | 20 | 0.10 | 0.10 | 0.924 | -0.08 | -0.08 | 0.937 |
| AB1-AP2 | 20 | -0.72 | -0.68 | 0.504 | 1.73 | 1.78 | 0.090 |
| AB1-B45 | 20 | **-6.24** | **-5.91** | **<0.001** | -0.28 | -0.29 | 0.779 |
| AB1-B61 | 20 | **-7.08** | **-6.70** | **<0.001** | 0.31 | 0.32 | 0.752 |
| AB1-N38 | 20 | **-5.35** | **-5.06** | **<0.001** | -1.99 | -2.06 | 0.053 |
| AB1-R25 | 20 | -1.79 | -1.70 | 0.105 | -2.32 | -2.40 | 0.026 |
| AP2-B45 | 20 | **-5.52** | **-5.22** | **<0.001** | -2.00 | -2.07 | 0.052 |
| AP2-B61 | 20 | **-6.36** | **-6.02** | **<0.001** | -1.42 | -1.46 | 0.159 |
| AP2-N38 | 20 | **-4.63** | **-4.38** | **<0.001** | **-3.72** | **-3.84** | **0.001** |
| AP2-R25 | 20 | -1.07 | -1.02 | 0.321 | **-4.05** | **-4.18** | **<0.001** |
| B45-B61 | 20 | -0.84 | -0.80 | 0.435 | 0.59 | 0.61 | 0.552 |
| B45-N38 | 20 | 0.89 | 0.84 | 0.409 | -1.72 | -1.77 | 0.091 |
| B45-R25 | 20 | **4.44** | **4.21** | **<0.001** | -2.05 | -2.12 | 0.047 |
| B61-N38 | 20 | 1.73 | 1.64 | 0.116 | -2.30 | -2.38 | 0.027 |
| B61-R25 | 20 | **5.29** | **5.00** | **<0.001** | -2.63 | -2.72 | 0.013 |
| N38-R25 | 20 | **3.55** | **3.36** | **0.003** | -0.33 | -0.34 | 0.736 |
