# Supplementary material for: Genetic variability of environmental sensitivity revealed by phenotypic variation in body weight and (its) correlations to physiological and behavioral traits
Source: PLoS One. 2017 Dec 18;12(12):e0189943. doi: 10.1371/journal.pone.0189943 (PMC5734726; doi:10.1371/journal.pone.0189943)
Supplement: S2 Table — (DOCX) [file pone.0189943.s003.docx]

**S2 Table Risk taking behaviour data (mean ± SEM) for 10 rainbow trout isogenic lines.** RT: risk taking. RT_%_time_spent: average percentage of time spent in the risky zone; RT_ntpass: average number of passages through the opening. SEM: standard error of the mean. ^(1)^ Statistical tests are from Millot *et al*. 2014.

| **Line** | **RT_%_time_spent** | **RT_ntpass** |
| --- | --- | --- |
| **A02h** | 28.55 (2.59) | 1.76 (0.20) |
| **A03h** | 51.44 (2.12) | 2.62 (0.14) |
| **A22h** | 34.97 (2.67) | 2.03 (0.17) |
| **A36h** | 50.72 (2.18) | 4.34 (0.44) |
| **AB1h** | 42.46 (2.39) | 2.63 (0.18) |
| **AP2h** | 35.44 (2.34) | 2.83 (0.23) |
| **B45h** | 46.10 (2.24) | 5.89 (0.42) |
| **B61h** | 45.27 (2.33) | 5.89 (0.55) |
| **N38h** | 49.69 (2.55) | 2.56 (0.16) |
| **R25h** | 47.01 (2.79) | 2.96 (0.32) |
|  |  |  |
| **Test^(1)^** | F_9,2630_=2.18, p<0.05 | F_9,2627_=38.47, p<0.001 |
