# Supplementary material for: Genetic variability of environmental sensitivity revealed by phenotypic variation in body weight and (its) correlations to physiological and behavioral traits
Source: PLoS One. 2017 Dec 18;12(12):e0189943. doi: 10.1371/journal.pone.0189943 (PMC5734726; doi:10.1371/journal.pone.0189943)
Supplement: S3 Table — (DOCX) [file pone.0189943.s004.docx]

**S3 Table Flight response and spatial exploration data (mean ± SEM) for 7 rainbow trout isogenic lines.** FR: flight response; SPE: spatial exploration. FR_Dist_Seq23: average distance travelled by each fish during Sequences 2 and 3 (after stimulus fall, in m); FR_Dist_diff21: difference in distance travelled between Sequences 2 and 1, in response to the stimulus fall (in m); SPE_Seq1_Z1 to Z4: average proportion of time spent by a fish in each zone during Sequence 1 (before the stimulus fall); SPE_Avg23_Z1 to Z4: average proportion of time spent by a fish in each zone during Sequences 2 and 3 (after the stimulus fall). SEM: Standard Error of the Mean. ^(1)^ Statistical tests are from Millot *et al*. 2014.

| **Line** | **FR_Dist_**  **Seq23** | **FR_Dist_**  **diff21** | **SPE_Seq1_**  **Z1** | **SPE_Seq1_**  **Z2** | **SPE_Seq1_**  **Z3** | **SPE_Seq1_**  **Z4** | **SPE_Avg23_**  **Z1** | **SPE_Avg23_**  **Z2** | **SPE_Avg23_**  **Z3** | **SPE_Avg23_**  **Z4** |
| --- | --- | --- | --- | --- | --- | --- | --- | --- | --- | --- |
| **A02h** | 367.3 (63.0) | -10.6 (26.0) | 27.6 (5.2) | 38.7 (4.3) | 29.5 (4.7) | 4.2 (1.3) | 25.3 (4.9) | 42.1 (4.9) | 28.8 (4.0) | 3.8 (0.8) |
| **A03h** | 54.3 (20.4) | -34.4 (14.7) | 10.7 (4.6) | 40.1 (9.0) | 34.3 (8.2) | 15.0 (8.3) | 1.8 (1.5) | 31.2 (11.7) | 47.3 (12.2) | 19.6 (10.1) |
| **AB1h** | 169.1 (45.9) | -31.7 (10.8) | 20.1 (5.9) | 25.9 (4.0) | 48.0 (7.8) | 5.9 (1.9) | 15.9 (4.9) | 35.1 (6.9) | 45.2 (8.8) | 3.8 (1.2) |
| **AP2h** | 206.3 (67.7) | -30.4 (16.3) | 13.5 (4.8) | 38.9 (8.3) | 41.2 (8.8) | 6.4 (2.5) | 15.8 (4.3) | 41.8 (9.4) | 37.3 (9.2) | 5.1 (2.4) |
| **B45h** | 90.8 (30.8) | -85.0 (19.9) | 26.0 (8.0) | 21.7 (3.6) | 32.2 (6.1) | 20.0 (10.3) | 26.4 (7.0) | 32.4 (7.2) | 22.4 (4.7) | 18.8 (10.4) |
| **B61h** | 113.0 (28.5) | -82.4 (20.5) | 26.4 (7.3) | 25.6 (1.4) | 43.0 (6.2) | 5.0 (1.6) | 28.5 (8.0) | 27.5 (6.9) | 38.9 (8.2) | 5.1 (2.8) |
| **R25h** | 212.2 (38.4) | -14.3 (13.9) | 23.5 (5.7) | 40.0 (6.1) | 31.6 (6.2) | 4.9 (2.1) | 22.4 (4.5) | 34.5 (6.3) | 35.6 (7.6) | 7.4 (4.4) |
|  |  |  |  |  |  |  |  |  |  |  |
| **Test^(1)^** | F_6,188_=6.42, p<0.001 | F_6,125_=3.44, p<0.01 |  |  |  |  |  |  |  |  |
