# Supplementary material for: Genetic variability of environmental sensitivity revealed by phenotypic variation in body weight and (its) correlations to physiological and behavioral traits
Source: PLoS One. 2017 Dec 18;12(12):e0189943. doi: 10.1371/journal.pone.0189943 (PMC5734726; doi:10.1371/journal.pone.0189943)
Supplement: S4 Table — (DOCX) [file pone.0189943.s005.docx]

**S4 Table Correlations between line means of CV body weight, behavioural traits (risk taking) and cortisol levels for 10 isogenic lines.** CV_indoor, CV_outdoor: coefficient of variation for body weight for 2 periods of time; RT_%_time_spent: average percentage of time spent in the risky zone; RT_ntpass: average number of passages through the opening; Chalg1_cort_S, Chalg2_cort_S and Chalg3_cort_S: post-stress cortisol levels for each of three confinement challenges; Cond_fact_D5 and Cond_fact_D15: condition factor at dates D5 and D15; Weight_D5 and Weight_D15: average body weight at dates D5 and D15; Weight_indoor and Weight_outdoor: average body weight for 2 periods of time. n.s.: P>0.10. In bold, P<0.010 (critical value for 79 tests for B-Y method Benjamini and Yekutieli 2001). Positive correlations are highlighted in green; negative correlations in orange.

|  | **CV_indoor** | **CV_outdoor** | **RT_%_time**  **_spent** | **RT_**  **ntpass** | **Chalg1_cort_S** | **Chalg2_cort_S** | **Chalg3_cort_S** | **Cond_fact_D5** | **Cond_fact_D15** | **Weight_D5** | **Weight_D15** | **Weight_indoor** |
| --- | --- | --- | --- | --- | --- | --- | --- | --- | --- | --- | --- | --- |
| **CV_outdoor** | 0.019 |  |  |  |  |  |  |  |  |  |  |  |
|  | *(n.s.)* |  |  |  |  |  |  |  |  |  |  |  |
| **RT_%_time_spent** | 0.38 | 0.55 |  |  |  |  |  |  |  |  |  |  |
|  | *(n.s.)* | *(n.s.)* |  |  |  |  |  |  |  |  |  |  |
| **RT_ntpass** | 0.70 | -0.22 | 0.44 |  |  |  |  |  |  |  |  |  |
|  | *(0.023)* | *(n.s.)* | *(n.s.)* |  |  |  |  |  |  |  |  |  |
| **Chalg1_cort_S** | 0.51 | 0.06 | 0.53 | 0.71 |  |  |  |  |  |  |  |  |
|  | *(n.s.)* | *(n.s.)* | *(n.s.)* | *(0.022)* |  |  |  |  |  |  |  |  |
| **Chalg2_cort_S** | -0.08 | 0.60 | 0.64 | 0.04 | 0.09 |  |  |  |  |  |  |  |
|  | *(n.s.)* | *(0.069)* | *(0.048)* | *(n.s.)* | *(n.s.)* |  |  |  |  |  |  |  |
| **Chalg3_cort_S** | -0.31 | -0.62 | -0.26 | 0.27 | -0.00 | -0.31 |  |  |  |  |  |  |
|  | *(n.s.)* | *(0.054)* | *(n.s.)* | *(n.s.)* | *(n.s.)* | *(n.s.)* |  |  |  |  |  |  |
| **Cond_fact_D5** | -0.30 *(n.s.)* | -0.62 *(0.057)* | -0.64 *(0.048)* | 0.19 *(n.s.)* | -0.05 *(n.s.)* | -0.39 *(n.s.)* | 0.73 *(0.016)* |  |  |  |  |  |
| **Cond_fact_D15** | -0.50 *(n.s.)* | 0.10 *(n.s.)* | -0.29 *(n.s.)* | -0.19 *(n.s.)* | 0.11 *(n.s.)* | -0.09 *(n.s.)* | 0.05 *(n.s.)* | 0.47 *(n.s.)* |  |  |  |  |
| **Weight_D5** | -0.16 *(n.s.)* | -0.39 *(n.s.)* | 0.02 *(n.s.)* | -0.11 *(n.s.)* | -0.16 *(n.s.)* | 0.25 *(n.s.)* | 0.20 *(n.s.)* | -0.10 *(n.s.)* | -0.41 *(n.s.)* |  |  |  |
| **Weight_D15** | -0.13 *(n.s.)* | -0.18 *(n.s.)* | -0.53 *(n.s.)* | -0.08 *(n.s.)* | -0.08 *(n.s.)* | -0.40 *(n.s.)* | 0.08 *(n.s.)* | 0.49 *(n.s.)* | 0.63 *(0.049)* | -0.16 *(n.s.)* |  |  |
| **Weight_indoor** | -0.19 *(n.s.)* | 0.32 *(n.s.)* | 0.46 *(n.s.)* | -0.24 *(n.s.)* | -0.10 *(n.s.)* | 0.73 *(0.016)* | -0.16 *(n.s.)* | -0.51 *(n.s.)* | -0.34 *(n.s.)* | 0.72 *(0.019)* | -0.41 *(n.s.)* |  |
| **Weight_outdoor** | -0.13 *(n.s.)* | -0.24 *(n.s.)* | -0.56 *(0.091)* | -0.13 *(n.s.)* | -0.18 *(n.s.)* | -0.30 *(n.s.)* | -0.03 *(n.s.)* | 0.43 *(n.s.)* | 0.54 *(n.s.)* | 0.01 *(n.s.)* | **0.96 *(<0.001)*** | -0.30 *(n.s.)* |
