# Supplementary material for: Genetic variability of environmental sensitivity revealed by phenotypic variation in body weight and (its) correlations to physiological and behavioral traits
Source: PLoS One. 2017 Dec 18;12(12):e0189943. doi: 10.1371/journal.pone.0189943 (PMC5734726; doi:10.1371/journal.pone.0189943)
Supplement: S5 Table — (DOCX) [file pone.0189943.s006.docx]

**S5 Table Correlations between line means of CV body weight, behavioural traits (spatial exploration SPE, flight response FR, risk taking RT) and cortisol levels for 7 isogenic lines.** CV_indoor and CV_outdoor: coefficient of variation for body weight for 2 periods of time; FR_Dist_Seq23: average distance travelled by each fish during Sequences 2 and 3 (after stimulus fall); FR_Dist_diff21: difference in distance travelled between Sequences 2 and 1, in response to the stimulus fall; SPE_Seq1_Z1 to Z4: average proportion of time spent by a fish in each zone during Sequence 1 (before the stimulus fall); SPE_Avg23_Z1 to Z4: average proportion of time spent by a fish in each zone during Sequences 2 and 3 (after the stimulus fall); RT_%_time_spent: average percentage of time spent in the risky zone; RT_ntpass: average number of passages through the opening; Chalg1_cort_S, Chalg2_cort_S and Chalg3_cort_S: post-stress cortisol levels for each of three confinement challenges; Cond_fact_D5 and Cond_fact_D15: condition factor at dates D5 and D15; Weight_D5 and Weight_D15: average body weight at dates D5 and D15; Weight_indoor and Weight_outdoor: average body weight for 2 periods of time. n.s.: P>0.10. In bold, P<0.009 (critical value for 136 tests for B-Y method Benjamini and Yekutieli 2001). Strong positive correlations are highlighted in green; strong negative correlations in orange.

|  | **CV_**  **indoor** | **CV_**  **outdoor** | **FR_Dist_**  **Seq23** | **FR_Dist_**  **diff_21** | **SPE_**  **Seq1_**  **Z1** | **SPE_**  **Seq1_**  **Z2** | **SPE_**  **Seq1_**  **Z3** | **SPE_**  **Seq1_**  **Z4** | **SPE_**  **Avg23_**  **Z1** | **SPE_**  **Avg23_**  **Z2** | **SPE_**  **Avg23_**  **Z3** | **SPE_**  **Avg23_**  **Z4** | **RT_**  **%_time_**  **spent** | **RT_**  **ntpass** |
| --- | --- | --- | --- | --- | --- | --- | --- | --- | --- | --- | --- | --- | --- | --- |
| **CV_**  **outdoor** | -0.01 |  |  |  |  |  |  |  |  |  |  |  |  |  |
|  | *(n.s.)* |  |  |  |  |  |  |  |  |  |  |  |  |  |
| **FR_Dist_**  **Seq23** | -0.49 | -0.21 |  |  |  |  |  |  |  |  |  |  |  |  |
|  | *(n.s.)* | *(n.s.)* |  |  |  |  |  |  |  |  |  |  |  |  |
| **FR_Dist_**  **diff_21** | **-0.88** | 0.27 | 0.66 |  |  |  |  |  |  |  |  |  |  |  |
|  | ***(0.009)*** | *(n.s.)* | *(n.s.)* |  |  |  |  |  |  |  |  |  |  |  |
| **SPE_**  **Seq1_Z1** | 0.46 | -0.09 | 0.39 | -0.27 |  |  |  |  |  |  |  |  |  |  |
|  | *(n.s.)* | *(n.s.)* | *(n.s.)* | *(n.s.)* |  |  |  |  |  |  |  |  |  |  |
| **SPE_**  **Seq1_Z2** | -0.57 | 0.35 | 0.42 | 0.81 | -0.47 |  |  |  |  |  |  |  |  |  |
|  | *(n.s.)* | *(n.s.)* | *(n.s.)* | *(0.027)* | *(n.s.)* |  |  |  |  |  |  |  |  |  |
| **SPE_**  **Seq1_Z3** | -0.15 | -0.53 | -0.28 | -0.22 | -0.24 | -0.42 |  |  |  |  |  |  |  |  |
|  | *(n.s.)* | *(n.s.)* | *(n.s.)* | *(n.s.)* | *(n.s.)* | *(n.s.)* |  |  |  |  |  |  |  |  |
| **SPE_**  **Seq1_Z4** | 0.43 | 0.24 | -0.66 | -0.52 | -0.19 | -0.34 | -0.31 |  |  |  |  |  |  |  |
|  | *(n.s.)* | *(n.s.)* | *(n.s.)* | *(n.s.)* | *(n.s.)* | *(n.s.)* | *(n.s.)* |  |  |  |  |  |  |  |
| **SPE_**  **Avg23_Z1** | 0.48 | -0.37 | 0.37 | -0.36 | **0.93** | -0.47 | -0.12 | -0.25 |  |  |  |  |  |  |
|  | *(n.s.)* | *(n.s.)* | *(n.s.)* | *(n.s.)* | ***(0.003)*** | *(n.s.)* | *(n.s.)* | *(n.s.)* |  |  |  |  |  |  |
| **SPE_**  **Avg23_Z2** | -0.71 | -0.37 | 0.80 | 0.69 | -0.09 | 0.51 | -0.19 | -0.36 | -0.01 |  |  |  |  |  |
|  | *(0.077)* | *(n.s.)* | *(0.030)* | *(0.088)* | *(n.s.)* | *(n.s.)* | *(n.s.)* | *(n.s.)* | *(n.s.)* |  |  |  |  |  |
| **SPE_**  **Avg23_Z3** | -0.39 | 0.21 | -0.33 | 0.27 | -0.67 | 0.26 | 0.59 | -0.27 | -0.72 | -0.25 |  |  |  |  |
|  | *(n.s.)* | *(n.s.)* | *(n.s.)* | *(n.s.)* | *(0.097)* | *(n.s.)* | *(n.s.)* | *(n.s.)* | *(0.068)* | *(n.s.)* |  |  |  |  |
| **SPE_**  **Avg23_Z4** | 0.40 | 0.50 | -0.69 | -0.38 | -0.30 | -0.10 | -0.43 | **0.94** | -0.40 | -0.44 | -0.11 |  |  |  |
|  | *(n.s.)* | *(n.s.)* | *(0.087)* | *(n.s.)* | *(n.s.)* | *(n.s.)* | *(n.s.)* | ***(0.002)*** | *(n.s.)* | *(n.s.)* | *(n.s.)* |  |  |  |
| **RT_%_time_**  **spent** | 0.38 | 0.58 | **-0.88** | -0.42 | -0.28 | -0.23 | 0.08 | 0.51 | -0.35 | -0.85 | 0.37 | 0.65 |  |  |
|  | *(n.s.)* | *(n.s.)* | ***(0.009)*** | *(n.s.)* | *(n.s.)* | *(n.s.)* | *(n.s.)* | *(n.s.)* | *(n.s.)* | *(0.016)* | *(n.s.)* | *(n.s.)* |  |  |
| **RT_**  **ntpass** | **0.92** | -0.21 | -0.59 | **-0.96** | 0.40 | -0.76 | 0.13 | 0.43 | 0.51 | -0.69 | -0.36 | 0.31 | 0.43 |  |
|  | ***(0.005)*** | *(n.s.)* | *(n.s.)* | ***(0.001)*** | *(n.s.)* | *(0.046)* | *(n.s.)* | *(n.s.)* | *(n.s.)* | *(0.083)* | *(n.s.)* | *(n.s.)* | *(n.s.)* |  |
| **Chalg1_**  **cort_S** | 0.66 | 0.28 | -0.69 | -0.64 | -0.00 | -0.31 | -0.37 | 0.83 | 0.04 | -0.54 | -0.37 | 0.83 | 0.66 | 0.68 |
|  | *(n.s.)* | *(n.s.)* | *(0.087)* | *(n.s.)* | *(n.s.)* | *(n.s.)* | *(n.s.)* | *(0.021)* | *(n.s.)* | *(n.s.)* | *(n.s.)* | *(0.021)* | *(n.s.)* | *(0.094)* |
| **Chalg2_**  **cort_S** | -0.23 | 0.56 | -0.03 | 0.33 | 0.02 | 0.27 | -0.18 | -0.17 | -0.01 | -0.15 | 0.11 | -0.02 | 0.44 | -0.12 |
|  | *(n.s.)* | *(n.s.)* | *(n.s.)* | *(n.s.)* | *(n.s.)* | *(n.s.)* | *(n.s.)* | *(n.s.)* | *(n.s.)* | *(n.s.)* | *(n.s.)* | *(n.s.)* | *(n.s.)* | *(n.s.)* |
| **Chalg3_**  **cort_S** | -0.11 | -0.78 | -0.19 | -0.33 | -0.12 | -0.55 | 0.80 | -0.06 | 0.12 | 0.07 | 0.09 | -0.32 | -0.10 | 0.27 |
|  | *(n.s.)* | *(0.040)* | *(n.s.)* | *(n.s.)* | *(n.s.)* | *(n.s.)* | *(0.030)* | *(n.s.)* | *(n.s.)* | *(n.s.)* | *(n.s.)* | *(n.s.)* | *(n.s.)* | *(n.s.)* |
| **Cond_fact_D5** | -0.04 *(n.s.)* | -0.83 *(0.020)* | 0.59 *(n.s.)* | -0.07 *(n.s.)* | 0.51 *(n.s.)* | -0.28 *(n.s.)* | 0.18 *(n.s.)* | -0.39 *(n.s.)* | 0.71 *(0.075)* | 0.54 *(n.s.)* | -0.56 *(n.s.)* | -0.64 *(n.s.)* | -0.77 *(0.044)* | 0.12 *(n.s.)* |
| **Cond_fact_D15** | -0.44 *(n.s.)* | 0.66 *(n.s.)* | 0.55 *(n.s.)* | 0.75 *(0.051)* | 0.07 *(n.s.)* | 0.65 *(n.s.)* | -0.72 *(0.068)* | -0.13 *(n.s.)* | -0.17 *(n.s.)* | 0.40 *(n.s.)* | -0.11 *(n.s.)* | 0.05 *(n.s.)* | -0.22 *(n.s.)* | -0.69 *(0.083)* |
| **Weight_D5** | -0.47 *(n.s.)* | -0.43 *(n.s.)* | 0.11 *(n.s.)* | 0.17 *(n.s.)* | -0.09 *(n.s.)* | -0.13 *(n.s.)* | 0.58 *(n.s.)* | -0.39 *(n.s.)* | 0.11 *(n.s.)* | 0.25 *(n.s.)* | 0.15 *(n.s.)* | -0.52 *(n.s.)* | -0.09 *(n.s.)* | -0.08 *(n.s.)* |
| **Weight_D15** | -0.02 *(n.s.)* | 0.16 *(n.s.)* | 0.57 *(n.s.)* | 0.18 *(n.s.)* | 0.76 *(0.046)* | -0.22 *(n.s.)* | -0.31 *(n.s.)* | -0.19 *(n.s.)* | 0.51 *(n.s.)* | 0.17 *(n.s.)* | -0.43 *(n.s.)* | -0.26 *(n.s.)* | -0.39 *(n.s.)* | -0.15 *(n.s.)* |
| **Weight_indoor** | -0.46 *(n.s.)* | 0.29 *(n.s.)* | -0.12 *(n.s.)* | 0.36 *(n.s.)* | -0.27 *(n.s.)* | 0.20052 *(n.s.)* | 0.25 *(n.s.)* | -0.25 *(n.s.)* | -0.23 *(n.s.)* | -0.07 *(n.s.)* | 0.41 *(n.s.)* | -0.16 *(n.s.)* | 0.41 *(n.s.)* | -0.22 *(n.s.)* |
| **Weight_outdoor** | -0.11 *(n.s.)* | 0.04 *(n.s.)* | 0.72 *(0.068)* | 0.28 *(n.s.)* | 0.77 *(0.041)* | -0.13 *(n.s.)* | -0.29 *(n.s.)* | -0.35 *(n.s.)* | 0.57 *(n.s.)* | 0.33 *(n.s.)* | -0.46 *(n.s.)* | -0.43 *(n.s.)* | -0.55 *(n.s.)* | -0.22 *(n.s.)* |

**S5 Table – continued**

|  | **Chalg1_cort_S** | **Chalg2_cort_S** | **Chalg3_cort_S** | **Cond_fact_D5** | **Cond_fact_D15** | **Weight_D5** | **Weight_D15** | **Weight_indoor** |
| --- | --- | --- | --- | --- | --- | --- | --- | --- |
| **Chalg2_cort_S** | 0.17 |  |  |  |  |  |  |  |
|  | *(n.s.)* |  |  |  |  |  |  |  |
| **Chalg3_cort_S** | -0.11 | -0.20 |  |  |  |  |  |  |
|  | *(n.s.)* | *(n.s.)* |  |  |  |  |  |  |
| **Cond_fact_D5** | -0.33 *(n.s.)* | -0.30 *(n.s.)* | 0.53 *(n.s.)* |  |  |  |  |  |
| **Cond_fact_D15** | -0.23 *(n.s.)* | 0.33 *(n.s.)* | -0.78 *(0.037)* | -0.28 *(n.s.)* |  |  |  |  |
| **Weight_D5** | -0.26 *(n.s.)* | 0.42 *(n.s.)* | 0.75 *(0.053)* | 0.43 *(n.s.)* | -0.34 *(n.s.)* |  |  |  |
| **Weight_D15** | -0.32 *(n.s.)* | -0.02 *(n.s.)* | -0.30 *(n.s.)* | 0.28 *(n.s.)* | 0.51 *(n.s.)* | -0.19 *(n.s.)* |  |  |
| **Weight_indoor** | -0.03 *(n.s.)* | 0.88 *(0.010)* | 0.20 *(n.s.)* | -0.23 *(n.s.)* | 0.06 *(n.s.)* | 0.72 *(0.067)* | -0.26 *(n.s.)* |  |
| **Weight_outdoor** | -0.43 *(n.s.)* | -0.01 *(n.s.)* | -0.24 *(n.s.)* | 0.43 *(n.s.)* | 0.51 *(n.s.)* | -0.08 *(n.s.)* | **0.98 *(<0.001)*** | -0.22 *(n.s.)* |
